# Supplementary material for: Genome-wide identification, characterization and gene expression of BES1 transcription factor family in grapevine (Vitis vinifera L.)
Source: Sci Rep. 2023 Jan 5;13:240. doi: 10.1038/s41598-022-24407-y (PMC9816167; doi:10.1038/s41598-022-24407-y)
Supplement: Supplementary file 3 — Supplementary Information. [file 41598_2022_24407_MOESM3_ESM.zip › Vvi_Atr/Vitis_vinifera.PN40024.v4.dna_sm.toplevel.fa.vs.Amborella_trichopoda.AMTR1.0.dna_sm.toplevel.fa.html/Atr-AmTr_v1.0_scaffold00186.html]

|  |  |  |  |  |  |  |  |  |  |  |  |  |  |
| --- | --- | --- | --- | --- | --- | --- | --- | --- | --- | --- | --- | --- | --- |
| Duplication depth | Reference chromosome | Collinear blocks | | | | | | | | | | | |
| 0 | Atr-ERN04722 |  |  |  |  |  |  |  |  |  |  |  |  |
| 0 | Atr-ERN04723 |  |  |  |  |  |  |  |  |  |  |  |  |
| 0 | Atr-ERN04724 |  |  |  |  |  |  |  |  |  |  |  |  |
| 0 | Atr-ERN04725 |  |  |  |  |  |  |  |  |  |  |  |  |
| 0 | Atr-ERN04726 |  |  |  |  |  |  |  |  |  |  |  |  |
| 0 | Atr-ERN04727 |  |  |  |  |  |  |  |  |  |  |  |  |
| 0 | Atr-ERN04728 |  |  |  |  |  |  |  |  |  |  |  |  |
| 0 | Atr-ERN04729 |  |  |  |  |  |  |  |  |  |  |  |  |
| 0 | Atr-ERN04730 |  |  |  |  |  |  |  |  |  |  |  |  |
| 0 | Atr-ERN04731 |  |  |  |  |  |  |  |  |  |  |  |  |
| 0 | Atr-ERN04732 |  |  |  |  |  |  |  |  |  |  |  |  |
| 0 | Atr-ERN04733 |  |  |  |  |  |  |  |  |  |  |  |  |
| 0 | Atr-ERN04734 |  |  |  |  |  |  |  |  |  |  |  |  |
| 0 | Atr-ERN04735 |  |  |  |  |  |  |  |  |  |  |  |  |
| 0 | Atr-ERN04736 |  |  |  |  |  |  |  |  |  |  |  |  |
| 0 | Atr-ERN04737 |  |  |  |  |  |  |  |  |  |  |  |  |
| 0 | Atr-ERN04738 |  |  |  |  |  |  |  |  |  |  |  |  |
| 0 | Atr-ERN04739 |  |  |  |  |  |  |  |  |  |  |  |  |
| 0 | Atr-ERN04740 |  |  |  |  |  |  |  |  |  |  |  |  |
| 0 | Atr-ERN04741 |  |  |  |  |  |  |  |  |  |  |  |  |
| 0 | Atr-ERN04742 |  |  |  |  |  |  |  |  |  |  |  |  |
| 0 | Atr-ERN04743 |  |  |  |  |  |  |  |  |  |  |  |  |
| 0 | Atr-ERN04744 |  |  |  |  |  |  |  |  |  |  |  |  |
